# Force-Generation by the Trans-Envelope Tol-Pal System

**DOI:** 10.3389/fmicb.2022.852176

**Published:** 2022-03-03

**Authors:** Melissa N. Webby, Daniel P. Williams-Jones, Cara Press, Colin Kleanthous

**Affiliations:** Department of Biochemistry, University of Oxford, Oxford, United Kingdom

**Keywords:** Tol-Pal, proton motive force, cell envelope, Gram-negative bacteria, force transduction, outer membrane

## Abstract

The Tol-Pal system spans the cell envelope of Gram-negative bacteria, transducing the potential energy of the proton motive force (PMF) into dissociation of the TolB-Pal complex at the outer membrane (OM), freeing the lipoprotein Pal to bind the cell wall. The primary physiological role of Tol-Pal is to maintain OM integrity during cell division through accumulation of Pal molecules at division septa. How the protein complex couples the PMF at the inner membrane into work at the OM is unknown. The effectiveness of this trans-envelope energy transduction system is underscored by the fact that bacteriocins and bacteriophages co-opt Tol-Pal as part of their import/infection mechanisms. Mechanistic understanding of this process has been hindered by a lack of structural data for the inner membrane TolQ-TolR stator, of its complexes with peptidoglycan (PG) and TolA, and of how these elements combined power events at the OM. Recent studies on the homologous stators of Ton and Mot provide a starting point for understanding how Tol-Pal works. Here, we combine *ab initio* protein modeling with previous structural data on sub-complexes of Tol-Pal as well as mutagenesis, crosslinking, co-conservation analysis and functional data. Through this composite pooling of *in silico*, *in vitro*, and *in vivo* data, we propose a mechanism for force generation in which PMF-driven rotary motion within the stator drives conformational transitions within a long TolA helical hairpin domain, enabling it to reach the TolB-Pal complex at the OM.

## Introduction

The cell envelope of Gram-negative bacteria is a three-layered structure that protects the cell and enables survival in changing, often hostile environments. The asymmetric outer membrane (OM) has an outer leaflet of lipopolysaccharide (LPS) and an inner leaflet of phospholipids that exclude both hydrophilic and hydrophobic molecules (Nikaido, 2003). The OM is not an energized membrane, thus active processes at the OM require energy input from either ATP hydrolysis in the cytoplasm or coupling to the proton motive force (PMF). One such active process is constriction of the OM during cell division. As bacterial cells divide, the cell envelope must be remodeled, a process that involves the Tol-Pal system (Gerding et al., 2007). Tol-Pal spans the cell envelope exploiting the PMF to coordinate separation of daughter cells and facilitate accumulation of the peptidoglycan (PG)-binding lipoprotein Pal at division sites, which provides stabilization of the OM during division (Lazzaroni et al., 1999; Cascales et al., 2000; Gerding et al., 2007; Egan, 2018; Petiti et al., 2019; Szczepaniak et al., 2020a; Yakhnina and Bernhardt, 2020). In addition to its normal physiological roles, Tol-Pal is exploited by group A bacteriocins and filamentous bacteriophages to promote their entry into Gram-negative bacteria (Nagel de Zwaig and Luria, 1967; Sun and Webster, 1986; Cascales et al., 2007; Kleanthous, 2010).

Seven genes are encoded within the *E. coli* Tol-Pal operon, including, *ybgC, tolQ, tolR, tolA, tolB, pal*, and *ybgF/cpoB* (Sturgis, 2001). However, the core components required for function comprise TolQ, TolR, TolA, TolB, and Pal. Perturbing this system by mutation and modulation of the expression of core genes confers a deleterious *tol* phenotype characterized by OM blebbing, cell-chaining, increased detergent/bile salt sensitivity, alteration of OM lipid ratios, and loss of pathogenicity (Bernadac et al., 1998; Szczepaniak et al., 2020b). Although the effects of *tol-pal* mutations are well-documented [reviewed in Szczepaniak et al. (2020b)], how force is transduced across the cell envelope is largely unknown despite a multitude of functional and biochemical studies on the individual proteins.

Recent data have suggested that a mobilization-and-capture mechanism underpins Tol-Pal function. The basis of the mechanism relies on force-dependent accumulation of Pal at division sites, where PG binding maintains cell wall interactions. Concentration of Pal at division sites prevents OM blebbing, a phenotype that is often observed in *tol-pal* mutants (Weigand et al., 1976; Weigand and Rothfield, 1976; Fung et al., 1978, 1980). This mechanism was first proposed by Szczepaniak et al. (2020b) who analyzed Pal-diffusion rates, showing that, in non-dividing cells, Pal diffuses slowly while in dividing cells Pal diffusion is accelerated (Connolley et al., 2021). Both the PMF and all components of the Tol-Pal system are required for these effects. In the proposed mechanism, TolB in the outer-periplasm (i.e., near the outer membrane) binds Pal to interrupt binding to PG (Bonsor et al., 2009), thereby increasing Pal diffusion in the OM. Following mobilization of Pal by TolB, the complex is recruited to the division site, where TolQ-TolR-TolA complexes accumulate (Gerding et al., 2007; Petiti et al., 2019). At the divisome, diffusing TolB-Pal complexes are captured by the TolQ-TolR-TolA complex and, in a force-dependent manner, Pal is released from TolB by TolA. Establishment of the TolA-TolB complex results in spatial segregation of TolB from Pal through translocation to the inner periplasm, where it is then released and free to diffuse and recycle. The force that drives this capture-release mechanism is hypothesized to rely on proton flux through the TolQ-TolR stator, which drives conformational changes in TolR and TolA in the periplasm.

To transmit force across the cell envelope, the TolQ-TolR-TolA complex likely transitions through a series of conformational states. This conformational plasticity hinders structural studies and is compounded by a lack of direct functional assays for TolQ-TolR-TolA activity *in vitro*. Nonetheless, structures for isolated domains of TolR (Parsons et al., 2008; Wojdyla et al., 2015) and TolA (Witty et al., 2002; Deprez et al., 2005; Lorenz et al., 2011; Ford et al., 2012; Li et al., 2012) from different species, in conjunction with structures of stator complexes from homologous systems, principally ExbB-ExbD (Celia et al., 2016, 2019; Hu et al., 2021) and MotA-MotB (Deme et al., 2020), provide insights into how the TolQ-TolR-TolA complex assembles. ExbB-ExbD is the inner membrane component of the Ton system, whilst MotA-MotB is the analogous complex in the Mot system. Like the Tol-Pal system, the Ton system and Mot system are protein complexes that span the cell envelope, transducing energy from the PMF across the inner membrane to facilitate nutrient import across the OM, and flagellum rotation, respectively. A multitude of structures for the Mot system components make this the best characterized of the three systems that are likely to function through a conserved mechanism. Unlike the Ton and Tol-Pal systems where a single copy of the stator complex is hypothesized to interact with periplasmic components TonB and TolA, respectively, multiple copies of the MotA-MotB stator complex interact in series with a large multimeric ring (the C-ring). Rotation of MotA about the stationary, PG-bound MotB, drives rotation of the flagellum via synchronized rotation of the C-ring (Deme et al., 2020; Johnson et al., 2021). Here, we review current structural and mutational data for TolQ-TolR-TolA and apply molecular modeling and co-conservation analysis to evaluate Tol-Pal function in the context of structural models. We propose a speculative mechanism to describe how the Tol-Pal system may utilize the PMF to transduce force to the OM during mobilization-and-capture of Pal.

## TolQ Forms a Pentameric Pore in the Inner Membrane

**FIGURE 1:**
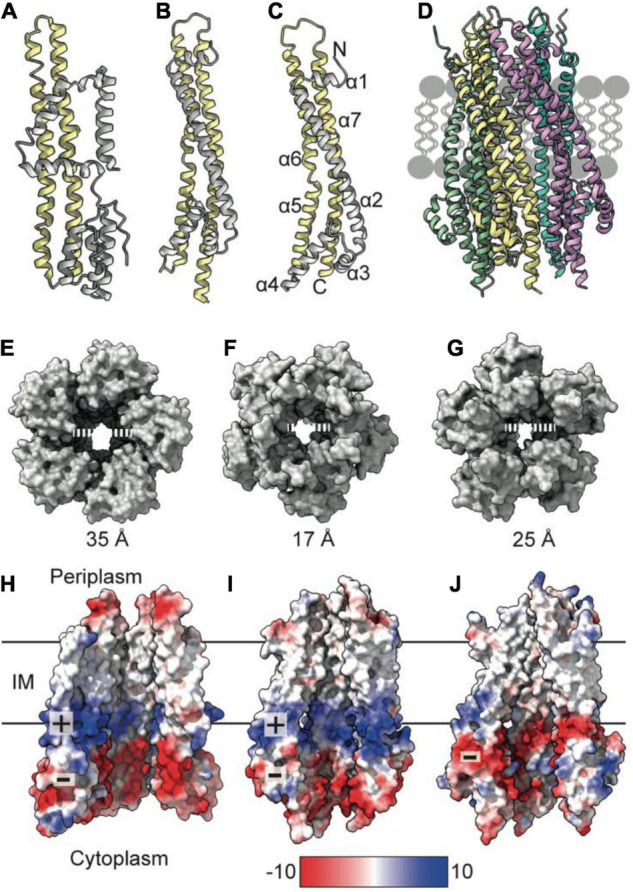
TolQ forms a pentameric pore. **(A)** MotA monomer transmembrane helices pack together to form a pentameric pore (*yellow*) and accessory helices are located on the exterior of the pore (*gray*). **(B)** ExbB monomer colored as in panel **(A)**. **(C)** The TolQ monomer (*E. coli* AlphaFold model) colored as in panel **(A)**, adopts a structure like that observed for ExbB. **(D)** Pentameric TolQ complex was generated following symmetry expansion of the AlphaFold monomer and energy minimization using RosettaRelax. Each monomer is colored separately. **(E–G)** The MotA, ExbB, and TolQ pentamers viewed from the cytoplasm to show the outward movement of helices that generate an expanded pore. By contrast, the structures can be overlaid almost perfectly at the periplasmic side. **(H–J)** A cut through the electrostatic surface of the MotA, ExbB, and TolQ pentamers, respectively, showing that the cytoplasmic side of the pore has a region of negative charge that is conserved across all three systems. A band of positive charge that spans the center of the pore is only observed in MotA and ExbB.

To understand mechanisms of force generation through the Tol-Pal system, we first reviewed current data for the IM stator complex formed by TolQ and TolR. Structural, biophysical, and native mass spectrometry data are consistent with stator complexes, including TolQ-TolR and homologs ExbB-ExbD and MotA-MotB, adopting a 5:2 stoichiometry (Celia et al., 2016, 2019; Deme et al., 2020; Santiveri et al., 2020). Recent structures of ExbB-ExbD and MotA-MotB solved by single-particle cryo-electron microscopy (EM) show that both ExbB and MotA form a conserved pentameric pore structure, suggesting that TolQ forms an analogous assembly. The monomeric structures of ExbB (244 amino acids in *E. coli*) and MotA (295 amino acids in *E. coli*) show that these proteins are predominantly helical. The first 100 amino acids at the N-terminus of these proteins form a variable number of short helices (Figures 1A,B). The remaining residues extend toward the C-terminus and assemble into two longer helices that span the membrane (Figures 1A,B). These transmembrane helices pack together to assemble the pentameric pore. The AlphaFold (Jumper et al., 2021) model of TolQ (230 amino acids in *E. coli*; Figure 1C) adopts an identical structural arrangement to that of ExbB (RSMD = 2.5 Å in Pymol), suggesting that the pentamers assemble in similar manner. We generated a TolQ pentamer through symmetry expansion of the AlphaFold monomer and energy minimization of the model using RosettaRelax (Khatib et al., 2011; Maguire et al., 2021; Figure 1D). The global assembly of the TolQ pore resembled the previously reported homology model (Szczepaniak et al., 2020b), although the *ab initio* model generated with Rosetta shows a distortion of helices that results in a wider pore relative to the homology model. Comparison of the pentameric structures for MotA, ExbB, and TolQ (Figures 1E–G) shows that the pore sizes on the periplasmic side of the pentamers are almost identical (∼25 Å). However, the diameter of the cytoplasmic pore is much wider in MotA (35 Å) relative to those of TolQ and ExbB, which are 25 and 17 Å, respectively (Figures 1E–G). Whether these differences have any physiological impact is unknown. The electrostatic profile of the interior of the MotA and ExbB pores comprises a region of positive charge spanning the middle section of the lumen, which sits directly above a band of negative charge (Figures 1H–I). In contrast, TolQ has only a single electronegative region spanning the lower section of the pore (Figure 1J). Across all structures, the location of the electronegative band within the pore is conserved, suggesting that electrostatics likely play a role in stator function.

## TolR Forms a Dimer That Inserts Within the TolQ Pore

**FIGURE 2:**
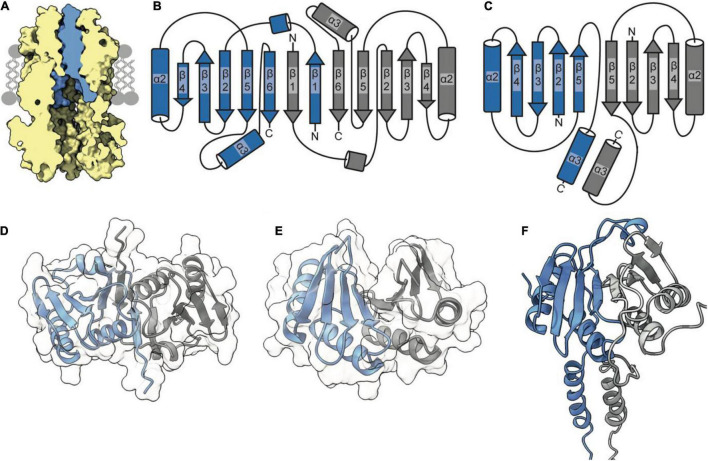
The TolR dimer has two distinct conformations. **(A)** The periplasmic entrance to the TolQ (*yellow*) pore is blocked by the transpore helices of the TolR dimer (*blue*). The TolQ-TolR model shown was assembled following alignment of the AlphaFold TolR monomer with the truncated TolR dimer from *H. influenzae* (TolR^H^). The full-length TolR dimer was then docked into the TolQ pentamer previously generated (Figure 1) based on position of ExbD in the ExbBD complex structure (Celia et al., 2019). The final TolQ-TolR complex shown was determined following energy minimization using RosettaRelax. **(B)** A depiction of the secondary structure of TolR from *E. coli* (TolR^E^) highlighting the strand-swapped dimer formed by β1 and β6 of each monomer. **(C)** A depiction of the secondary structure of TolR^H^ has a dimer interface formed through interactions of β5-β5. **(D)** The TolR^E^ strand-swapped dimer (PDB ID 5BY4) solved by X-ray crystallography shows the 180° rotation of one monomer (*gray*) relative to the other (*blue*). **(E)** The TolR^H^ structure solved by NMR (PDB ID 2JWK) is in an open PG-binding conformation. Each monomer is represented in *blue* and *gray*, respectively. **(F)** The *E. coli* TolR dimer, generated in Rosetta, models the entirety of the protein, which adopts an open conformation like that of TolR^H^.

The periplasmic side of the stator pore in both MotA and ExbB is blocked by two helices (Celia et al., 2019; Deme et al., 2020) contributed by the dimer of MotB and ExbD, respectively. Assuming that the TolQR complex adopts an analogous 5:2 structure, it is likely to assemble in a similar manner, with the TolR dimer inserting within the pentameric pore of TolQ (Figure 2A). These transpore helices (TPHs) that bind TolQ are connected through a disordered linker sequence to the periplasmic domain of TolR, which binds to PG (Wojdyla et al., 2015). It is thought that the periplasmic domain of TolR extends and contracts via this disordered sequence to allow TolR to bind both TolQ and PG simultaneously. Structures of the periplasmic domain of the TolR dimer from both *E. coli* (TolR^E^) and *H. influenzae* (TolR^H^), respectively, have been solved to reveal that two distinct dimer conformations are adopted (Figures 2B–E). Because of the high degree of sequence conservation (60%) and identical secondary structure predictions, it is unlikely that the unique dimer conformations are species specific. Rather, it has been proposed that these two conformations represent different functional states, one capable of binding PG and one in which PG binding is occluded (Wojdyla et al., 2015).

The concept of transitioning between binding-competent and binding-incompetent conformations has also been proposed for MotB, where truncations of the N-terminal domain are thought to be associated with rearrangement of the C-terminal helix to occlude the PG-binding site (Roujeinikova, 2008). Later studies showed that it is the unfolding of the N-terminal helix that results in extension of the disordered linker sequence (Kojima et al., 2009; O’Neill et al., 2011). This extension allows MotB to reach and bind PG, stabilizing the complex and allowing protons to access the exposed MotA channel. This model suggests that rearrangement of the dimer to modulate PG binding might be a mechanism that is conserved across these molecular motors.

Analysis of the dimer structures for TolR^E^ and TolR^H^ reveals that, in the TolR^E^ structure solved by X-ray crystallography (Figure 2D), TolR monomers are rotated ∼180° relative to each other, thereby obliterating the proposed PG binding cleft due to strand swapping (Figures 2B,D). In contrast, the TolR^H^ dimer solved by NMR (Parsons et al., 2008) adopts an open conformation (Figure 2D), with a cleft appearing between the two monomers in which PG is proposed to bind (Figure 2E). Like MotB, the TolR^H^ structure was obtained by truncating N- and C-terminal sequences that are otherwise present in the TolR^E^ structure; 22 residues at the N-terminus form a short helix (residues 51–58) and β-strand (residues 81–86), while at the C-terminus 10 residues form another β-stand (residues 135–139). It is β-strand 1 of TolR^E^ that is involved in generation of the strand-swapped dimer. To acquire more information regarding state transitions, we used Rosetta to generate a model of the full-length *E. coli* TolR dimer (Figure 2F), starting from the monomer predicted by AlphaFold (Jumper et al., 2021). During model building the movement of the N-terminal TPH of TolR, which is involved in binding TolQ (Figure 2A), was more restricted than that of the periplasmic domain. The dimer model produced from Rosetta adopted a PG-binding conformation similar to that of TolR^H^ (Figure 2F).

To generate the TolR^E^ strand-swapped conformation from our TolR model, the dimer interface formed by β5-β5 interactions must be broken, and the β-sheet spanning residues 81–86 (β1) must form. In addition, one monomer must rotate ∼180° relative to the other (Figure 2D), which can only be achieved through breaking the dimer interface and moving the TPH upward (∼10–15 Å) out of the TolQ pore. Secondary structure predictions of *E. coli* TolR predict that the sequence corresponding to β6 has a high propensity to form a β-strand. However, residues 81–86 surrounding β1 are predicted to be disordered. Based on these secondary structure predictions and structural data, β1 and β6 are likely transient structures. We suggest that a strand-swapped dimer could form if β1 and β6 were present and if the TPHs were not fixed within the TolQ pore. Further experimental evidence is required to determine if TolR transitions between PG-binding competent and occluded states, or if these states are artifacts induced by sequence truncation and crystallization.

## Mutagenesis and Co-Conservation Analysis Corroborate Insertion of TolR Into the TolQ Pore

**FIGURE 3:**
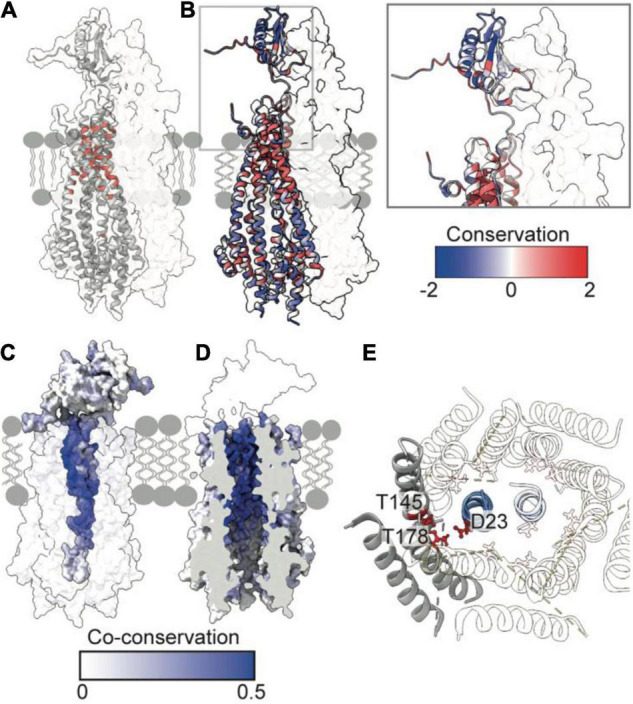
Essential, conserved, and co-conserved stator residues map to the TolQR lumen. **(A)** *Red* identifies essential residues that confer membrane instability when substituted (Vianney et al., 1994; Goemaere et al., 2007; Zhang et al., 2011). Two TolQ monomers are shown in cartoon format with a single TolR monomer. **(B)** The relative conservation of TolQ-TolR residues, which was generated following alignment (Clustal Omega) of ten TolQ sequences from different species, indicates that the most conserved residues map to the top or bottom of the TolQ lumen. The most-conserved residues of TolR map to the transpore helix and β-strand two. **(C,D)** The TolQ-TolR co-conservation profile of individual residues as scored by RaptorX suggest that the TolR helix inserts into the center of the TolQ pore. Co-conserved TolR and TolQ residues are predominantly within the transpore helix and lumen, respectively. **(E)** A ring of highly conserved threonine residues (*red sticks*) within the transmembrane region of TolQ (T145 in helix 6 and T178 in helix 7) are proposed to stabilize protonated TolR D23 (*red sticks*), based on structures of the MotA-MotB and ExbB-ExbD complexes (Celia et al., 2019; Deme et al., 2020).

Recent structures of ExbB-ExbD and MotA-MotB provide insight into how the N-termini of dimeric ExbD and MotB bind within their respective pentameric pores. Using Rosetta and models of ExbB-ExbD and MotA-MotB as a guide, we generated a model of the TolQ-TolR stator complex (Figure 3). Similar to MotB and ExbD, the single TPHs of the TolR dimer are arranged parallel to each other, effectively creating a plug that would prevent free diffusion of hydronium ions through TolQ. Interactions that support assembly of the complex are largely localized to the TPHs of TolR within the TolQ pore. The structured periplasmic domain of TolR is located 20 Å away from TolQ and does not form interactions with the pore. In the 10 top-scoring Rosetta models of TolR, the periplasmic domain is located at different distances from TolQ, while the remainder of the stator complex remains largely unperturbed. This situation likely reflects the dynamic nature of the disordered linker sequence connecting the periplasmic domain of TolR to the TPHs.

The reliability of the TolQ-TolR complex model was strengthened by demonstrating its consistency with previous mutation and functional data. Early studies, which assumed the TPH was transmembrane, focused on characterizing its position relative to the TolQ helices (Kampfenkel and Braun, 1993; Vianney et al., 1994; Zhang et al., 2009, 2011), and delineating potential proton-trafficking pathways by site-directed mutagenesis (Goemaere et al., 2007). The positions of mutated essential residues that yielded an OM instability phenotype map to the interior of the TolQ pore (Figure 3A). Moreover, these residues are predominantly located within the upper region of the TolQ pore that interacts with the TPHs of the TolR dimer.

To identify regions of functional importance independently, sequence conservation of TolQ-TolR across 10 different species was calculated and mapped onto the TolQ-TolR model (Figure 3B). As expected, the most extensive sequence conservation was observed within the lumen of the TolQ pentamer rather than its exterior. Highly conserved residues were clustered at the upper and lower extremities of the pore, corresponding to the binding site for the TolR TPHs and a conserved electronegative region within the pentameric pore complex (Figures 1J, 3B), respectively. For TolR, the most highly conserved residues mapped to the TPH and β2 (Figure 3B).

To differentiate between raw conservation and inter-subunit co-conservation, residue contact probabilities within the TolQ-TolR complex were scored with RaptorX (Zeng et al., 2018), and the highest pairwise score of individual residues mapped accordingly (Figures 3C,D). These computational data show that the residues with the highest co-conservation scores map to the luminal face of TolQ helix 7 (85% of the top 20 predicted contacts) and the TolR TPH (Figures 3C,D), supporting the proposed trans-pore insertion of TolR. The strongest co-conservation observed was between TolR V27-TolQ L175 (0.54), which are located on the same face within 9 Å of each other in our TolQ-TolR model. Similarly, the essential residue TolR D23, which is suggested to play a role in proton trafficking (Cascales et al., 2001; Goemaere et al., 2007), was highly co-conserved with TolQ T178 (0.50), and these residues are situated 4–5 Å of each other in the TolQ-TolR model (Figure 3E). TolQ T145 was previously suggested to play a role in PMF transduction (Goemaere et al., 2007), and it is also co-conserved with TolR D23, but with a lower probability (0.24) (Figure 3E). These aspartate and threonine residues are highly conserved across homologous stator systems (Braun and Herrmann, 1993), in accordance with the putative proton-conducting mechanisms proposed for MotA-MotB and ExbB-ExbD (Celia et al., 2019; Deme et al., 2020; Santiveri et al., 2020; Hu et al., 2021). TolR D23 is likely protonated (or coordinates a hydronium ion) and is stabilized by adjacent threonine residues before conformational changes result in rotation of TolQ relative to TolR, concomitant with proton release to the cytoplasm. As well as being both essential and co-conserved, the adjacent position of TolR D23 and TolQ T145/178 in the TolQ-TolR model, and their conservation across homologous stators, supports their proposed roles in proton trafficking and stabilization. Taken together, these sequence conservation and mutation analyses support structural models in which the TolR dimer inserts into the TolQ pentamer, and protonation of TolR D23 is stabilized by a conserved threonine ring in TolQ.

## TolA Forms an Elongated Helical Hairpin That Could Span the Periplasm

**FIGURE 4:**
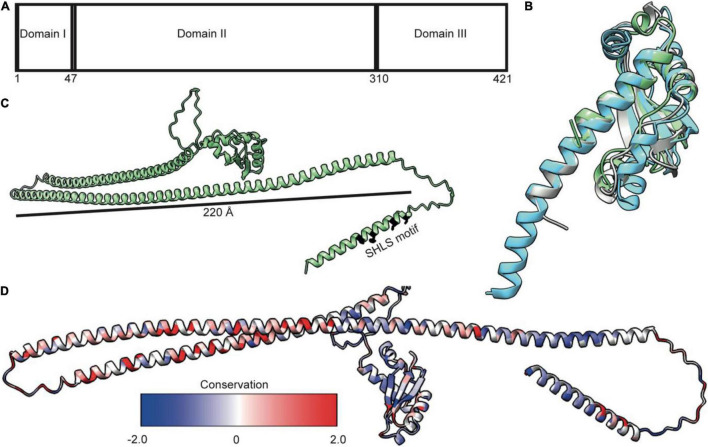
TolA is a largely helical protein with three distinct domains. **(A)** TolA contains three annotated domains: domain I is the transmembrane helix, domain II is an elongated helical hairpin, and domain III is a small globular protein. Numbering is based on the *E. coli* sequence. **(B)** Structural overlay of TolA III from *P. aeruginosa* (*cyan*, PDB ID 1LR0) and *V. cholerae* (*gray*, PDB ID 4G7X) with the equivalent domain from *E. coli* (*green*, PDB ID 1S62) (RMSD of 1.3 and 2.0, respectively) showing that, although poorly conserved (20 and 25%, respectively), the domains have near identical folds. **(C)** The AlphaFold model of TolA from *E. coli.* The central domain II is predicted to form a helical hairpin that is 220 Å in length, which is long enough to span the periplasm. **(D)** Sequences of nine TolA proteins from different species were aligned in Clustal Omega, and sequence conservation was mapped onto the *E. coli* TolA structure within ChimeraX. Conserved residues are largely located within the C-terminal end of the helical hairpin of domain II. Residues in domains I and III are less conserved.

TolA is essential for bridging the TolQ-TolR-TolA complex from the IM through the periplasm to the OM. By doing so, it generates a connection from the stator to TolB and its complex with Pal. TonB, which forms a complex with ExbB-ExbD, is functionally equivalent to TolA. Low sequence conservation, especially in the regions that are thought to span the periplasm, suggests TolA and TonB are analogs lacking a common ancestor (Braun and Herrmann, 1993), although this is disputed (Witty et al., 2002). TolA is divided into three distinct domains (TolA I-III); an N-terminal domain anchoring the complex in the IM (TolA I), an elongated domain which forms an extended structure that spans the periplasm (TolA II), and a third, globular domain required for interactions at the OM (TolA III) (Figure 4A; Levengood et al., 1991; Levengood-Freyermuth et al., 1993). Although only limited structural data exists for TolA I and TolA II, the structure of TolA III from *E. coli* has been solved both in isolation and in complex with binding partner peptides, including bacteriophage proteins and colicins (Lubkowski et al., 1999; Lorenz et al., 2011; Ford et al., 2012; Li et al., 2012). TolA III forms a compact domain made up of three β-strands and three α-helices (Figure 4B). This domain arrangement is conserved across several species (Figure 4B). The extended structure of *Pseudomonas aeruginosa* TolA III reveals that helix three extends further than is observed in the *E. coli* structure (Figure 4B). SAXS models of TolA II-III confirm the predicted elongated structure of TolA II and combined with sequence-based secondary structure predictions and circular dichroism data, indicates that this domain likely forms a helical hairpin (Levengood et al., 1991; Witty et al., 2002). Although *ab initio* models and SAXS data are consistent with TolA II existing as an extended helical hairpin, TolA II likely exhibits both a helical hairpin ground state and a PMF-energized state where TolA II extends through holes in the PG to reach the OM. The conformational transitions within TolA II have been shown to be PMF-dependent, with TolA I also required for energy transduction to the OM (Germon et al., 2001). This conclusion is supported by data that show tethering of TolA to the OM by colicins, bound at the OM but unable to translocate, restricts its diffusion in the inner membrane. However, upon abolition of the PMF, TolA exhibits unrestricted diffusion, suggesting that non-energized TolA cannot reach the OM (Rassam et al., 2018). Together, these data imply that energy dependent state transitions of TolA are an essential element of force transduction in Tol-Pal.

AlphaFold (Jumper et al., 2021) models of TolA from *P. aeruginosa* and *E. coli* (Figure 4C) show a single transmembrane helix within TolA I that is separated from TolA III by a helical hairpin that constitutes TolA II. The structures predicted by AlphaFold are consistent with the experimentally determined models for TolA III (Figures 4B,C). The hairpin structure of *P. aeruginosa* TolA II is 130 Å in length, whereas in *E. coli*, TolA II is 220Å in length, which is almost the entire width of the periplasm (250–300 Å) (Asmar et al., 2017). TolA sequences from nine Gram-negative bacteria show that most sequences are similar in the number of residues, with conserved residues located primarily within the helical hairpin structure of TolA II rather than in TolA I or TolA III (Figure 4D). This arrangement could suggest that TolA II interacts with other proteins through this domain, or that these residues are merely essential for ensuring that the helical hairpin structure forms to maintain the relative distance of TolA III from the IM. The C-terminal end of TolA II (residues 280–313) has previously been shown to bind the soluble periplasmic protein CpoB/YbgF; (Krachler et al., 2010). CpoB is not essential in *E. coli*, but its expression is tightly co-regulated within the *tol-pal* operon. CpoB binds TolA adjacent to the TolB-binding site of TolA III. It contains a number of so-called tetratricopeptide repeats (TPRs) that are typically found in protein-protein interaction hubs (D’Andrea and Regan, 2003). CpoB interacts with both TolA and the PG-synthase PBP1B (Gray et al., 2015), suggesting that TolA interactions with CpoB at the cell septum are temporally and spatially coordinated with the PG-remodeling complex PBP1B-LpoB. TolA was also found to interact with PBP1B directly, but this interaction was abrogated upon deletion of TolA I, suggesting that either energization is required for this interaction or that the binding site of PBP1B is within TolA I (Gray et al., 2015).

## TolQ-TolR-TolA Complex Assembly Suggests That the TolA Transmembrane Helix Inserts Into a TolQ Groove on the Exterior of the TolQ Pentamer

**FIGURE 5:**
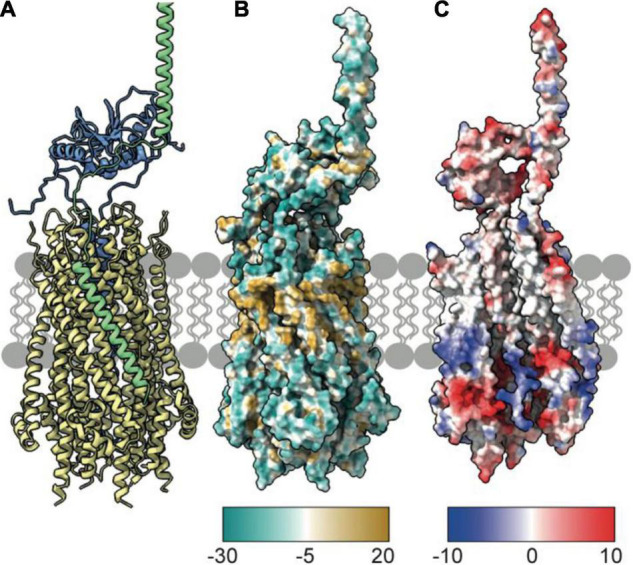
TolA packs against the TolQ pentamer to form the TolQRA complex. **(A)** The Rosetta model of the TolQ-TolR-TolA complex from *E. coli* was generated following placement of full length TolA, taken from the AlphaFold database, within 10 Å of the TolQ-TolR complex (Figure 2). A series of Rosetta docking, and refinement steps were run to generate the lowest energy model shown. TolA (*green*) packs against the TolQ pentamer (*yellow*) with the transmembrane helix of TolA placed in a parallel arrangement within the exterior helices of TolQ. The TolR dimer sits above TolQ with its transpore helices packed within the TolQ pore. The model suggests that TolR is in close proximity to the hairpin (domain II) of TolA. **(B)** Hydrophobicity map of the TolQ-TolR-TolA complex shows a hydrophobic (*gold*) belt around the middle of the TolQ-TolA complex. This depiction is consistent with this region residing in the IM. **(C)** Electrostatic properties of the TolQ-TolR-TolA complex are displayed with chains B and C from TolQ hidden to show the charge within the pore. The extreme N-terminus of TolR is electropositive, complementing the electronegative charge within the pore. A similar charge-coupling is seen between the transmembrane helix of TolA and the exterior of TolQ. The TolQ-TolR-TolA model is available as a PDB file from associated Supplementary Material.

It has been suggested that TolA I associates with TolQ-TolR via an SHLS motif (Figure 4C), which was first identified in TonB, where it is essential both for stator association and PMF transduction (Karlsson et al., 1993; Koebnik et al., 1993). The SHLS motif denotes a non-consecutive, four-residue sequence composed of two serines, a histidine, and a leucine residue, which are located in the transmembrane helix of TonB and TolA. These residues are well conserved across species and are essential for activity. In *E. coli* TolA, this motif is composed of residues S18, H22, L29 and S33. Based on homology with the Ton system, in which maintenance of the position of SHLS residues is essential for ExbB binding (Larsen and Postle, 2001), it is reasonable to assume that this motif plays a similar role in the TolQ-TolA complex. In TonB, the unilateral geometry of S16 and H20 in the SHLS motif is essential for function (Larsen et al., 1994, 1999; Germon et al., 2001; Larsen and Postle, 2001). To date, very limited structural information is available for the TolQ-TolR-TolA complex. We used Rosetta to generate a low-energy model of TolQ-TolR-TolA (Figure 5A). In this model, TolA I sits parallel to the helices of TolQ, packed within a groove on the exterior of the pentamer structure (Figure 5A). The hydrophobic profile of the exterior of the TolQ-TolR-TolA complex (Figure 5B) shows that the position of TolA relative to TolQ creates a continuous hydrophobic belt around the complex that would be amenable for insertion within the IM. The flexible N-terminal domain of TolR is anchored in the TolQ pore through electrostatic matching (Figure 5C) between the electronegative pore of TolQ and the electropositive charge at the extreme N-terminus of TolR. The position of TolA relative to TolQ and TolR also contributes some charge-matched regions (Figure 5C). It seems likely that charge complementarity plays a role in the assembly of the TolQ-TolR-TolA complex. Analogous charge matching is observed in both the Mot and Ton systems.

**FIGURE 6:**
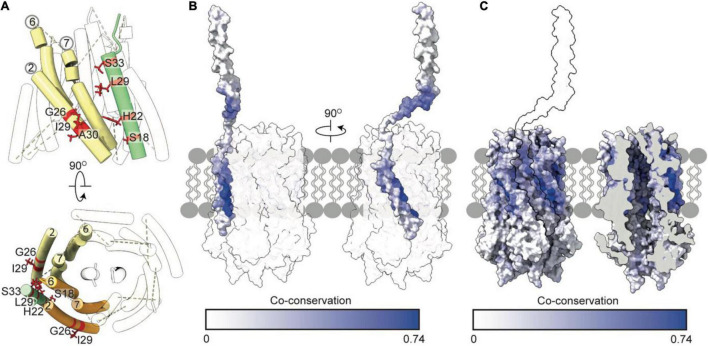
*Tol-pal* suppressor and co-conservation analysis suggest the transmembrane helix of TolA docks within a groove formed by two TolQ helices. **(A)** The organization of TolQ helices (numbered) relative to the TolA transmembrane helix (*green*) within the TolQ-TolA model. Individual TolQ monomers are labeled in *yellow* and *orange*, respectively. The position of the TolA SHLS motif and suppressor mutations identified previously in TolQ (G26, I29 and A30 helix 2, labeled *red*) (Germon et al., 1998). **(B,C)** Maximum co-conservation scores of TolQ-TolA from RaptorX output mapped onto the TolQ-TolA model. The face of the TolA transmembrane helix in contact with TolQ in the model is strongly co-conserved. The TolQ helix 2 groove shows high co-conservation with the transmembrane helix of TolA (*yellow* outline).

Our model of TolQ-TolR-TolA suggests that TolA I is oriented parallel to the TolQ helices, placing the SHLS motif between adjacent TolQ monomers (Figure 6A). In previous suppressor mutagenesis studies exploiting inactive TolA SHLS mutations, TolQ suppressor mutants were identified at G26, I29 and A30, respectively (Germon et al., 1998). The suppressor mutation sites are adjacent to TolA I within the model (Figure 6A). A co-conservation analysis between TolQ and truncated TolA (residues 1–100) yielded the highest scores for residues on the face of TolA I that are in contact with TolQ helix 2 (Figures 6B,C), corroborating the predicted position of TolA I. As expected, residues within the SHLS motif were highly co-conserved. The top twenty predicted inter-residue contacts were found exclusively within the hydrophobic region of TolQ helix 2, suggesting that this region constitutes the TolQ-TolA binding interface. TolA I also exhibits co-conservation with the TolQ pore-forming helices 6–7; cysteine-scanning data suggest that these helices undergo dynamic, PMF-dependent conformational rearrangements (Zhang et al., 2011), which might indicate transient contacts with TolA during TolQ rotation.

## TolB Interacts With TolA and Pal at the Outer Membrane

Force-transduction by the TolQ-TolR-TolA complex through the cell envelope has thus far been demonstrated only for the import of the bacteriocin colicin E9 (Francis et al., 2021), not for its physiological function of dissociating the TolB-Pal complex. TolB is a soluble periplasmic protein that interacts with TolA, Pal and colicins (Clavel et al., 1998; Walburger et al., 2002; Loftus et al., 2006). TolB has two domains, an N-terminal α/β domain and a C-terminal six-bladed β-propeller domain (Loftus et al., 2006; Bonsor et al., 2009; Zhang et al., 2010; Szczepaniak et al., 2020a). Interactions with binding partners requires conformational cycling between distinct states of TolB (Bonsor et al., 2007, 2009), which favor protein specific binding. Here, we focus on TolB interactions with TolA and Pal.

TolA III interacts with the disordered N-terminus of TolB. Yeast two-hybrid studies are consistent with residues 301–421 of TolA III forming a complex with the N-terminal 119 amino acids of TolB (Walburger et al., 2002). More recently, the solution NMR structure of a TolAIII-TolB (residues 22–34) complex has been reported (Szczepaniak et al., 2020a). It revealed that the proteins interact through β-strand augmentation in which the C-terminal helix of TolA III is displaced, exposing the TolB-binding site. This interaction is analogous to that observed between TonB and the TonB boxes of TonB-dependent transporters (TBDTs). TBDT TonB boxes are short sequences of ∼8 residues that bind TonB through β-strand augmentation. Despite the low sequence conservation between TolA and TonB, the C-terminal domain of these proteins has the same fold and both exploit β-strand augmentation to facilitate binding to their respective partners.

When TolB is complexed with Pal, its N-terminus is buried, precluding interaction with TolA. Pal is a periplasmic protein attached to the inner leaflet of the OM through an N-terminal lipoyl tether. Like Lpp and OmpA, Pal binds to non-crosslinked stem peptides of PG, while simultaneously remaining bound to the OM. During cell division, Pal is recruited to division sites through interactions with TolB. TolB-binding and PG-binding sites overlap on Pal. Therefore, TolB association with Pal blocks its ability to bind PG, enhancing Pal diffusion in the OM and allowing for Pal recruitment to the division septum where TolQ-TolR-TolA is already localized (Bonsor et al., 2007; Szczepaniak et al., 2020a).

The structure of Pal is similar to that of other PG-binding proteins such as TolR, MotB, and ExbD, with a α/β sandwich fold where the PG- binding cleft is formed by the loops that connect elements of secondary structure. TolB is in conformational equilibrium between a compact state favored by its association with Pal, in which its N-terminus is buried between its two domains, and a relaxed state in which its N-terminus is free to bind TolA. A ternary TolA-TolB-Pal complex can also form, and it is on this complex that the TolQ-TolR stator is thought to exert force (Bonsor et al., 2007; Szczepaniak et al., 2020a,b). The release of Pal from TolB has been proposed to occur via a mechanism analogous to that of the unplugging of TBDTs by ExbB-ExbD-TonB. When TolA binds TolB, it effectively pulls TolB away from Pal to the inner periplasm through holes in the PG, thereby spatially segregating TolB from Pal to prevent immediate re-association. The role of TolB-Pal and TolB-TolA interactions in the function of the Tol-Pal system during cell division has been reviewed previously (Szczepaniak et al., 2020b).

## Proton Motive Force Transduction by TolQ-TolR-TolA Likely Facilitates TolA Extension

**FIGURE 7:**
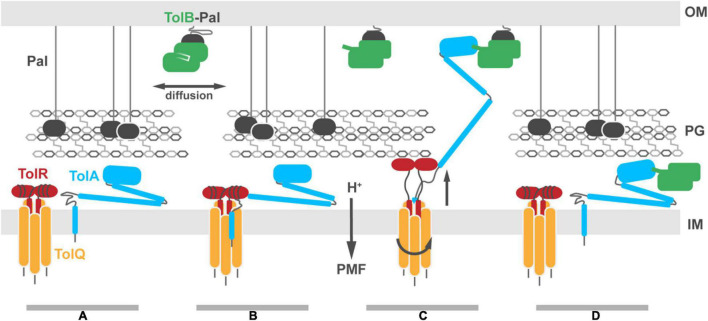
Putative model for Tol-Pal force transduction through the cell envelope. **(A)** The TolQ-TolR stator and TolA diffuse freely within the IM. The abundant OM lipoprotein Pal is predominantly bound to the PG, stabilizing its connection to the OM. A sub-population of Pal molecules are bound to TolB. This binding blocks association of those Pal molecules with PG, enhancing their diffusion in the OM. **(B)** TolA associates with the TolQ-TolR stator, inserting between monomers of TolQ. **(C)** In response to protonation of stator residues, TolR extends and binds the PG layer, allowing rotation of the TolQ helices, which in turn drives extension of the TolA helical hairpin through pores in the PG layer. The C-terminal domain of TolA associates with the N-terminus of TolB in its complex with Pal. **(D)** Deprotonation of stator residues drive retraction of the TolR periplasmic domain away from the PG. Relaxation of the TolA helical hairpin toward the IM could be either through coupling to this structural change or refolding of the hairpin, following dissociation of TolA from the stator (as shown in the panel). We speculate that retraction of the TolA hairpin provides the driving force for dissociating TolB-Pal complexes at the OM and pulling TolB below the PG layer. The figure does not show recruitment of the TolQ-TolR-TolA complex to the divisome which concentrates Pal deposition at division sites to stabilize the invaginating OM.

The mechanism by which the Tol-Pal system generates force to drive TolB-Pal dissociation at the OM remains elusive. It is likely that a series of sequential PMF-dependent conformational transitions are transmitted through the periplasm. The key proteins required for formation of an energy transducing complex are TolQ, TolR, TolA, TolB and Pal, the last of which is not directly energized. The stoichiometry of the TolQ-TolR-TolA complex has not been explicitly demonstrated but is likely to be similar to Mot and Ton (Figure 7A; Celia et al., 2016, 2019; Deme et al., 2020; Santiveri et al., 2020). The free diffusion of TolA in the IM (Gerding et al., 2007; Petiti et al., 2019; Szczepaniak et al., 2020a), suggests that multiple TolAs might bind a single stator complex. However, the estimated copy number of TolA (400–800 copies per cell) (Levengood et al., 1991) is 4–5× lower than that of TolR (Mueller et al., 1993), suggesting it is more likely that a single TolA is bound per stator complex (Figure 7B). TolA likely associates with the TolQ-TolR stator following 2D diffusion in the IM, with its long helical hairpin and globular domains (TolA II and TolA III) positioned below the PG (Figures 7A,B). This view is consistent with the observation that TolA-GFP exhibits unrestricted Brownian motion in the IM unless it forms a complex with the OM by reaching through the PG (Rassam et al., 2018). Our models and co-conservation analysis show that TolA could interact with both TolQ and TolR simultaneously to drive the initial assembly of the complex (Figure 7B). It is currently unclear whether TolA interacts with a strand-swapped dimer or an open PG-binding conformation of TolR. However, the PMF-dependent transition of TolR between these two dimer states is likely to be integral to force transduction.

Once the TolQ-TolR-TolA complex has formed, proton flux through the stator drives conformational changes in both TolR and TolA, possibly through a combination of rotary and piston-like motions. Protease-accessibility and immunoblotting experiments have suggested that TolA undergoes extensive structural rearrangements in response to the PMF. These arise from interactions of TolA I with the TolQ-TolR stator (Germon et al., 2001). Homologs, including MotA-MotB and ExbB-ExbD, are proposed to be rotary stator motors. Since TolQ is a related stator, we suggest that during rotation it could distort the geometry of TolA I, facilitating extension of the TolA II helical hairpin (Figure 7C). In conjunction with rotation of TolQ, TolR must also undergo conformational changes within the disordered sequence connecting the TPH to its periplasmic domain. The corresponding region in ExbD has been shown to undergo PMF-dependent conformational change suggesting force transduction facilitates conformational rearrangements of the periplasmic domain (Ollis et al., 2012). The electronegative charge at the cytoplasmic side of the TolQ lumen is not at an equivalent position around the lumen. Thus, as TolQ rotates, the electropositive N-terminus of TolR must rearrange to maintain charge matching between TolQ and TolR and to minimize charge repulsion between TolR monomers. This would result in an up-and-down piston motion that could propagate through TolR, resulting in an upward extension of the periplasmic domain that would allow for binding to the PG (Figure 7C). The distance from the disordered sequence connecting the TPH of TolR to the structured periplasmic domain is sufficient to reach the PG, ∼75 Å away. A similar extension has been proposed for MotB (Kojima et al., 2009; O’Neill et al., 2011).

If TolR exhibits a preference for binding non-crosslinked PG, it could bias the position of the TolQ-TolR stator toward pores in the PG. Assuming this were the case, TolR binding to PG would necessarily guide TolA II through the same hole during PMF-dependent extension (Figure 7C). Extended TolA would then be in a position for TolA III to bind TolB within the TolB-Pal complex at the OM (Figure 7C). The force-dependent step could then be either through PMF-driven deformation of TolA II by the TolQ-TolR stator or simple refolding of TolA II, following dissociation from the stator. Retraction of TolA II through the PG layer would bring TolB with it, thereby dissociating the TolB-Pal complex (Figure 7D).

Overall, the combination of new tools for predicting protein folds, modeling techniques, and integration of experimental data culminate in the model of TolQ-TolR-TolA presented here. This model supports the position of the TolR TPH within the lumen of the TolQ pentamer, and it includes a proposed TolQ-TolA interface within the plane of the IM, facilitated by charge and hydrophobicity matching. To integrate this model into the cell envelope and reconcile the PMF-dependence of both the conformational rearrangements of TolA and of the interactions of TolA with the OM, we posit that in its non-energized state TolA is localized below the PG layer, with PMF-energized TolQ-TolR required to guide TolA II to the outer periplasm, enabling TolA III to bind TolB-Pal in the OM.

## Outstanding Questions

Stabilization of the OM during cell division by Tol-Pal is facilitated by PMF-dependent structural rearrangements of the proteins involved. TolA exists predominantly in a compact state between the IM and the PG layer, a hypothesis based on structural and microscopy data (Rassam et al., 2018; Szczepaniak et al., 2020a). In this model, TolA does not reach the OM unless energized by the TolQ-TolR stator, which leads to conformational rearrangements in TolA II and extension across the periplasm through pores in the PG. Upon binding the N-terminus of TolB, the cycle reverses, returning TolA II to the inner membrane attached to its TolB cargo. Such a model invokes a multitude of further questions regarding the nature of the energized and non-energized states of TolA and how these structures are linked to the extension and retraction of the TolA II helical hairpin. Furthermore, there is the question of how the extension of TolA is coordinated with formation of the TolQ-TolR-TolA complex, with proton flux, and with the rotation of the TolQ-TolR stator.

Data on MotA-MotB and ExbB-ExbD suggest that the TolR dimer is the origin of rotation, but little can be inferred currently about how this rotation affects the conformation of TolA. The likely insertion of the TolA transmembrane helix into the groove between the outer TolQ helices begs additional questions of stoichiometry, affinity, and regulation. Does TolA play an active role as a receptor for proton flux, or is its energization a by-product of rotary motion and insertion between TolQ helices? Although H22 within TolA I has been suggested to be involved in the pathway of proton flux because it is essential for Tol-Pal function, based on our model it seems more likely that H22 simply contributes to the TolQ-binding interface. This is consistent with the Ton-system in which mutagenesis studies of H20 of the TonB SHLS motif suggest that the conserved histidine is of structural importance, rather than serving as a proton acceptor (Larsen et al., 2007; Postle et al., 2010; Swayne and Postle, 2011; Ollis et al., 2012). Consistent with the rotary motion of TolQ-TolA being important for function, *in vivo* fluorescence anisotropy experiments of N-terminal GFP-TonB fusions have highlighted restricted diffusion and PMF-dependent rotation of the TonB transmembrane helix (Jordan et al., 2013). This likely occurs because TonB binds ExbB in a manner analogous to TolA binding TolQ, suggesting that rotation of stator complexes is the cornerstone of the energization mechanism in both cases.

Our model assumes that TolR is the stationary component about which TolQ rotates, and that it is spatially and temporally fixed by its association with PG. This assumption raises the question of how PG binding is modulated. It is known that TolQ-TolR and TolA freely diffuse in the IM and are independently recruited to the septal ring (Gerding et al., 2007; Petiti et al., 2019; Szczepaniak et al., 2020a). This trafficking of TolQ-TolR and TolA to the septum depends on the -remodeling of PG and the activity of FtsN and its interactors (e.g., FtsWI) rather than on direct interaction of TolR and TolA with FtsN (Gerding et al., 2007; Hale et al., 2021). This suggests that the local composition of PG plays a role in TolQ-TolR-TolA localization, either through the function of intermediary trafficking proteins or the preferential retention of the complex through direct Tol-PG interactions.

The parallels between the Tol and Ton systems extend beyond the structural similarities of the stator complexes. The SHLS motif is present in both TolA and TonB, and exchange of this sequence motif between the effectors subsequently establishes interactions with the opposite stator complex (Braun and Herrmann, 1993). TonB has a central domain that is substantially different from TolA II; it is predicted to be unstructured and proline rich. This suggests that these effectors span the periplasm by different mechanisms yet interact with their respective stators in a homologous fashion. Characterizing how stator rotation facilitates conformational rearrangements in TolA and TolR represents the next step in understanding the mechanism by which this PMF-dependent system transduces force across the periplasm to drive stabilization of the OM.

## Data Availability Statement

The data supporting the findings of the study are available in the associated Supplementary Material and are available upon request from the corresponding author.

## Author Contributions

MW and DW-J: data acquisition. MW, DW-J, and CK: writing – original draft review and editing. All authors contributed to the article and approved the submitted version.

## Conflict of Interest

The authors declare that the research was conducted in the absence of any commercial or financial relationships that could be construed as a potential conflict of interest.

## Publisher’s Note

All claims expressed in this article are solely those of the authors and do not necessarily represent those of their affiliated organizations, or those of the publisher, the editors and the reviewers. Any product that may be evaluated in this article, or claim that may be made by its manufacturer, is not guaranteed or endorsed by the publisher.

## Supplementary Material

RaptorX Co-conservation files and model PDB files are available in Supplementary Material.
