# Supplementary figures and images for: Force-Generation by the Trans-Envelope Tol-Pal System
Source: Front Microbiol. 2022 Mar 3;13:852176. doi: 10.3389/fmicb.2022.852176 (PMC8928145; doi:10.3389/fmicb.2022.852176)

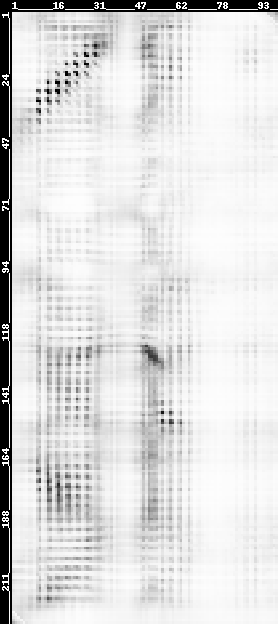

Supplement: Supplementary file 2 [file Data_Sheet_2.ZIP › Supplementary-RaptorX/TolQ(A)-TolA100(B).all_in_one/690377A_690377B.gcnn_inter.png]

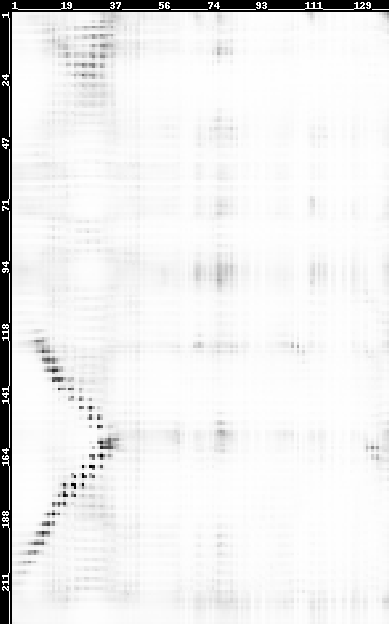

Supplement: Supplementary file 2 [file Data_Sheet_2.ZIP › Supplementary-RaptorX/TolQ(A)-TolR(B).all_in_one/688448A_688448B.gcnn_inter.png]
